# On Night Diet

**Published:** 1873-02

**Authors:** 


*On Night Diet.*

Dr. Friedreich Betz (Memorabilien xvi, 4 p. 92, 1872) remarks upon the subject of food as follows:

Very small children take food often, both day and night and at regular intervals, two or three hours apart. It is only later and gradually that the child begins to limit its meals to the daytime. Hunger is then experienced at certain times of the day; in the morning between 9 and 10, in the afternoon between 4 and 5 o'clock. At these periods the chief meals of the day should really be taken, but different circumstances have changed these times among most people. Food is then taken according to habit or time: whether this custom is perfectly safe or not must remain as yet an open question. At any rate the physiological periodicity of demand should be regarded as a main column in the temple of health.

But where digestion is rapid, where circumstances permit only smaller quantities, or where the body is changed by disease and the various metamorphoses are interfered with, there must be a change not only in the quantity and quality, but also in the time of administration. The exclusive ingestion of food by day is insufficient for the body in disease, and it becomes necessary, then, to feed the patient at night also. The manner in which it is to be taken, and the amount, are questions which may not be established by general law. Each case has its own requirements. Patients themselves do not often know how much they are reduced as inanition sometimes progresses very slowly, for instance, in chronic phthises; here, too, appetite becomes blunted. It is only by direct experimentation, thus, upon each case that the time, amount and frequency of food can be established. Yet it may be stated, in general terms, that the most appropriate time is between 9 and 10 in the evening and 1 and 3 in the morning. The character of the food is to be determined according to circumstances; it should be, in general, easily digestible food. Alcohol, opium and cod-liver oil may be of great utility at times.

The diseases which call for this supervision as to night food, are, besides the acute and chronic hemorrhages, bronchorrheas, suppurations, diarrheas, diabetic conditions, convalescence after exhausting diseases, inanition after nervous diseases, long continued hysterias, hypochondriases and other mental conditions; also in mental overwork, premature senility, inanition consecutive to chronic gastric catarrh, profuse sweatings, physical exhaustion, infantile atrophy, dropsies, etc.

Inanition often occurs so insidiously that the patient himself is not aware of it. A most particularly valuable symptom indicative of its invasion is sleeplessness, in combat with which, alcohol is a remedy not to be underestimated. It is certainly true that some reduced patients are more or less somnolent; these patients, too, require nocturnal food.—*Schmidt's Jahrbucher*, Nov. 12, '72. *The Clinic.*

---

*A Lumbricus Discharged through an Abscess about the Hip-Joint —Recovery.*

The following curious case occurred in the Mansfield Workhouse Infirmary in May last:

J. W., aged thirteen, was suffering from strumous disease of the hip-joint, but able to get about on crutches. The usual abscesses kept forming and bursting about the joint: one abscess, however, after bursting, discharged as part of its contents a large lumbricus, fully eighteen inches long, and coiled upon itself. How did it get there? At any rate, the wound healed rapidly, and the boy's health improved considerably.—*Lancet*, Nov. 30, 1872.

